# Developing a programme theory to explain how primary health care teams learn to respond to intimate partner violence: a realist case-study

**DOI:** 10.1186/s12913-015-0899-8

**Published:** 2015-06-09

**Authors:** Isabel Goicolea, Anna-Karin Hurtig, Miguel San Sebastian, Carmen Vives-Cases, Bruno Marchal

**Affiliations:** Unit of Epidemiology and Global Health, Department of Public Health and Clinical Medicine, Umea University, SE-90187 Umea, Sweden; Public Health Research Group, Department of Community Nursing, Preventive Medicine and Public Health and History of Science, Alicante University, Alicante, Spain; CIBER of Epidemiology and Public Health (CIBERESP), Barcelona, Spain; Department of Public Health, Institute of Tropical Medicine, Antwerp, Belgium

**Keywords:** Realist evaluation, Intimate partner violence, Primary health care teams, Team learning, Case study, Spain

## Abstract

**Background:**

Despite the progress made on policies and programmes to strengthen primary health care teams’ response to Intimate Partner Violence, the literature shows that encounters between women exposed to IPV and health-care providers are not always satisfactory, and a number of barriers that prevent individual health-care providers from responding to IPV have been identified. We carried out a realist case study, for which we developed and tested a programme theory that seeks to explain how, why and under which circumstances a primary health care team in Spain learned to respond to IPV.

**Methods:**

A realist case study design was chosen to allow for an in-depth exploration of the linkages between context, intervention, mechanisms and outcomes as they happen in their natural setting. The first author collected data at the primary health care center La Virgen (pseudonym) through the review of documents, observation and interviews with health systems’ managers, team members, women patients, and members of external services. The quality of the IPV case management was assessed with the PREMIS tool.

**Results:**

This study found that the health care team at La Virgen has managed 1) to engage a number of staff members in actively responding to IPV, 2) to establish good coordination, mutual support and continuous learning processes related to IPV, 3) to establish adequate internal referrals within La Virgen, and 4) to establish good coordination and referral systems with other services. Team and individual level factors have triggered the capacity and interest in creating spaces for team leaning, team work and therapeutic responses to IPV in La Virgen, although individual motivation strongly affected this mechanism. Regional interventions did not trigger individual and/ or team responses but legitimated the workings of motivated professionals.

**Conclusions:**

The primary health care team of La Virgen is involved in a continuous learning process, even as participation in the process varies between professionals. This process has been supported, but not caused, by a favourable policy for integration of a health care response to IPV. Specific contextual factors of La Virgen facilitated the uptake of the policy. To some extent, the performance of La Virgen has the potential to shape the IPV learning processes of other primary health care teams in Murcia.

**Electronic supplementary material:**

The online version of this article (doi:10.1186/s12913-015-0899-8) contains supplementary material, which is available to authorized users.

## Background

Men’s intimate partner violence (IPV) against women, defined as “*any behaviour within an intimate relationship that causes physical, sexual or psychological harm, including acts of physical aggression, sexual coercion, psychological abuse and controlling behaviours*”, is widespread and has devastating effects on the health and wellbeing of women and children [1–3]. The most recent global estimates of violence against women show that 35 % of women worldwide have experienced physical and/or sexual IPV or non-partner sexual violence [4]. Within the EU-27, between 20 % and 25 % of all women have experienced IPV at least once in their lifetime [5].

Health care services can play a key role in the prevention and management of IPV and its harmful health effects, because women suffering from IPV may use health care services more often than other public services. The health system- and especially first line health services- can be an IPV survivor’s first and only point of contact with professionals, and as such represents an opportunity for improved health and well-being [3, 6–8]. First-line primary health-care centres (PHCC) are the public institutions most frequently accessed by women exposed to IPV – more than legal, social services or the police. In addition, women exposed to IPV visit health-care professionals more often than women who are not exposed to IPV.

There is general consensus that the health sector should carry out the following actions: ask all women about violence, stay alert to possible signs and symptoms, provide health care assistance and register all cases, provide information on available resources, coordinate with other professionals and institutions, and provide evidence of the magnitude and seriousness of IPV. All these actions should be carried out while ensuring privacy and confidentiality, in a supportive environment where women’s experiences are validated and their decisions are respected [3]. Primary care IPV interventions have demonstrated patient-level benefits and economic evaluations indicate that they are cost-effective [9, 10].

However, the literature shows that encounters between women exposed to IPV and healthcare providers are not always satisfactory, and a number of barriers that prevent individual health-care providers from responding to IPV have been identified. These include organizational barriers, time constraints, an attitude of blaming vis-à-vis women exposed to IPV, lack of training, and lack of community resources to team up with, to cite just a few. Individual characteristics such as age, gender, and attitudes towards IPV have also been associated with the type and quality of response provided by health-care providers [11–14].

When it comes to PHCCs, team characteristics also influence the response, since in such services the individual provider-patient encounter is framed in interdisciplinary work and the relations between the different professionals and women. The extent to which a primary health care team learns to respond to IPV depends not only on the complexity of such intervention, but also on factors at the health care providers’ level, team level and the broader organizational and social context [15].

Research that explores why, how and under which circumstances primary health care teams learn to respond to IPV is, to the best of our knowledge, very scarce. Such studies could support the strengthening of interventions that are currently being carried out. This study uses a realist case study design to develop a programme theory that seeks to explain how, why and under which circumstances a primary health care team in Spain learned to respond to IPV.

## Methods

### A realist case study of how primary health care teams learn to respond to IPV

In this study, we used a realist evaluation approach. Realist evaluation is a type of theory driven evaluation that aims to ascertain why, how and under which circumstances programs succeed or fail. Realist evaluation begins with the formulation of the theory behind the development of an intervention, known as the *programme theory* (PT). The PT is formulated on the basis of previous research and/or knowledge and the experience of stakeholders involved in the intervention. The PT is then tested through empirical research and when sufficient evidence is generated, a middle-range theory can be developed, which provides plausible explanations of why, how and under what circumstances the intervention triggers mechanisms that lead to the desired outcomes [15–20]. This middle-range theory can then be tested and refined in a new cycle of realist evaluations.

**Fig. 1:**
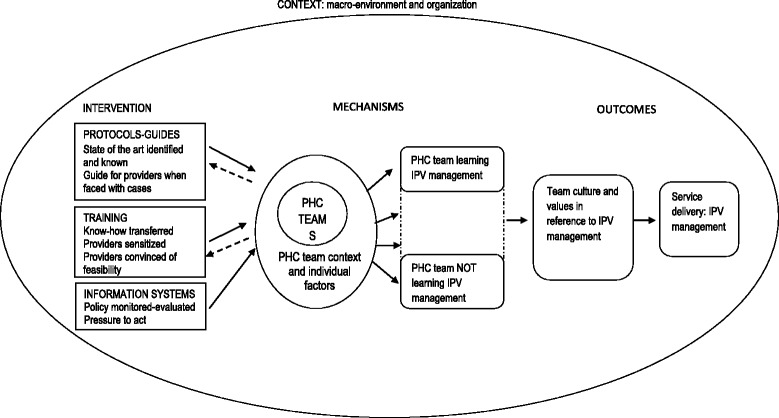
Conceptual framework for analysing the process of team learning of IPV management within primary health care facilities in Spain (modified from Goicolea I, Vives-Cases C, San Sebastian M, Marchal B, Kegels G, Hurtig AK: How do primary health care teams learn to integrate intimate partner violence (IPV) management? A realist evaluation protocol. Implement Sci 2013, 8:36. Doi:10.1186/1748-5908-8-36)

In a previous paper, we documented the development of the initial programme theory underlying the IPV policy in Spain [15] and how its implementation can be shaped through team learning at the level of primary health care teams. This initial programme theory - that will be referred to as PT1 from here onwards - emerged from literature review and exploratory interviews with the planners of the intervention, both at regional and national level. PT1 did not incorporate the views and perceptions of the providers who were expected to implement such interventions. PT1 stated that:

“Backed by the 2004 Gender Based Violence law, the Spanish health system began implementing actions to support primary health care teams’ learning about practices for IPV detection and management, and- to a lesser extent- prevention. These actions focused on: 1) developing protocols and guidelines to identify the state of the art knowledge and make it available to teams and providers, 2) training of health professionals, aimed at raising the awareness of providers, transferring know-how, and convincing them to respond to IPV, and 3) implementing systems to monitor the implementation of the policy and pressure those responsible for acting. These actions generated individual learning and team learning processes within primary health care teams. However, contextual factors related to the team and its individual members- including culture and values- strongly influenced how those interventions were adopted. They also influenced how team learning on IPV took place and, consequently, the response offered to women exposed to IPV.” The figure below presents the key elements of PT1 (Fig. 1).

Since PT1 was formulated essentially on the basis of initial exploratory research, we aimed at refining it through the in-depth study of one primary health care team before testing it in 4 other cases. This would allow us to capture implementation failure as well as better understand how individual and team level factors could influence the adoption of the interventions. With this aim, we conducted an exploratory single case study of a selected primary health-care team. We chose the primary health care team of La Virgen, located in the autonomous region of Murcia. We choose La Virgen as a critical case because it was considered by the regional health authorities to be a particularly positive case. This aligns with case study and realist evaluation’s principles of purposive sampling. Case studies are useful to explore how and why social phenomena work: this design indeed allows for an indepth exploration of the interrelationship of context, intervention, mechanisms and outcomes as they occur in their natural setting [21].

### The case: the PHCC “La Virgen”

La Virgen (LV) is located in a low- to middle-income neighbourhood of a medium-size city within the autonomous region of Murcia. The team consists of 14 family doctors, 14 nurses, 3 paediatricians, 2 midwifes, 1 physiotherapist, 1 social worker, 5 auxiliary nurses and 10 administrative staff. There is also a psychologist who works at the centre once per week. LV is a teaching centre, meaning that residents from the family medicine programme do practice during their training. In Spain the majority of primary health centres are also teaching centres.

Since La Virgen is based in the region of Murcia, it arguably has been influenced by the interventions carried out in this region aimed to improve the health-sector response to IPV. The region of Murcia has published a protocol that requests practitioners to document the woman’s history, to evaluate the risk of further IPV, to provide information to the woman on possible interventions, safety planning and referrals, to ensure non-judgemental and supportive attitudes, to avoid contacting the partner and referring to couple counselling, and to respect the woman’s autonomy. Detection is based on warning signs and conducting appropriate clinical inquiry when they are present. Routine screening for IPV is neither included in the protocol, nor promoted in this region [22]. From the La Virgen participants, 60 % stated that they have read the protocol, but during the interviews and observation it seemed that few actually used it. The Murcia region has prioritized training health professionals on IPV. Training has been ongoing for several years on a voluntary basis. In La Virgen, 16 % of professionals stated that they have attended a basic training on IPV (more than 20 h), 20 % advanced training (i.e. trainer of trainers, Master or Diplomas in related topics, etc.) and12 % of them stated that they have never received any training or participated in sensitizing campaigns.

In Murcia, as in most of the Spanish regional health systems, the systems for monitoring the response to IPV are weak. The staff at La Virgen have not received any feedback regarding their indicators for IPV since the policy was implemented.

In each of the PHCCs in Murcia, one health professional has been designated as IPV coordinator. This person serves as a link between the person in charge of the IPV programme in Murcia’s regional health system and the primary health care centre. The IPV coordinator also serves as a nexus between specialized services attending to IPV victims and PHC services. In La Virgen, the team decided that one of the nurses involved in IPV response would be designated as IPV coordinator. More information regarding the main characteristics of the interventions carried out in Murcia (and other Spanish regional health systems) to integrate a health-care response to IPV can be found in another paper (see [22]).

La Virgen health centre participated in a training programme on the *biopsychosocial approach to women’s malaise* that took place between 2003 and 2006. The women’s malaise approach considers that somatic symptoms with no identifiable organic cause are related to contextual, subjective and gender-related factors, and that a purely biomedical approach to health cannot adequately address such symptoms [23, 24]. The training encouraged providers to incorporate a biopsychosocial approach to health, and improved their competencies to implement such an approach during their consultations. A number of primary health care teams in Murcia and another province participated in this training, but only LV staff implemented the approach in their clinical practice.

The aim of the training was: 1) to identify psychosocial factors associated with clinical diagnosis and facilitate psychosocial changes in patients, 2) to achieve clinical improvement of somatic and emotional symptoms and decrease the use of psychotropic drugs, and 3) to reduce psychosocial discomfort [23, 24]. This training process also promoted a structure of monthly meetings to support the work of health professionals. These monthly meetings were moderated by an external psychologist and served as a forum where providers could discuss cases and improve their skills. The meetings were titled “the malaise group”, were held on Fridays, starting early in the morning and lasting for one and a half hours. The malaise group meetings began with the training on women’s malaise, but later evolved to focus on the discussion of IPV. The social worker described how the meetings developed from being a small group of friends gathering in her office to becoming monthly meetings, in which around 20 professionals – from a total of 35 - participated on a regular basis. They usually followed the structure of “clinical case” discussions, but focusing on cases on IPV. In practice, one of the health professionals presents a case from her practice and how she responded to it. The group comments and may give suggestions. Sometimes, they invite external experts to present issues related with IPV or the IPV coordinator or the social worker will present new initiatives launched by Murcia’s health system in reference to IPV.

Another activity carried out in La Virgen was the “women’s group”. The social worker began the women’s group in 2007, and since then, several women have participated. The women’s group gathered weekly for four hour long sessions and that was open to women who were referred by LV’s health providers or the social worker if they had non-specific complaints associated with women’s malaise. Women from different ages, ethnic backgrounds and professions participated. In the group, women practiced chi kung, metamorphic massage and talk therapy. Women participated in the group meetings for eight months, after which they were “discharged”. Some of them continued gathering outside the health centre in what they called “the community group”, meeting in a secondary school in the neighbourhood. Even if IPV was not the focus of the group, many disclosed suffering from IPV and collected the courage to do this because of the therapeutic process they went through by participating in the community group.

### Data collection

We collected both qualitative and quantitative data. The first author visited LV between January and March 2013 and collected qualitative data through document review, interviews and observation. Collection of multiple sources of data is a requirement and a strength of the exploratory case studies such as the one we carried out in La Virgen since it allows to triangulate across data sources.

Interviews were conducted with 17 team members in LV. In order to maximize the variety of informants and get a broader picture, different professionals were included (social workers, family doctors, paediatricians, nurses, midwifes, auxiliary nurses, administrators, nurse coordinators, medical coordinators, and the staff members in charge of IPV); professionals interested in the issue of IPV, those not interested and one who was perceived by the team as more critical were also interviewed. One of the approached professionals refused to participate. In order to obtain insights in the ‘external’ perspective, 5 members from services outside the health centre who offered social, legal and psychological support to women exposed to IPV. Finally, we also interviewed 4 women who had suffered IPV and were using the health service. Due to the fact that respondents gave great importance to the training programme on women’s malaise, the leader of the programme was also interviewed.

Interview guides were developed, containing questions related to aspects of PT1, such as team learning, team culture, adoption of protocols, training programmes and monitoring systems. However, care was taken to remain open for any other relevant information. All interviews were recorded.

The interview setting and time was adapted to the needs of the interviewees, and with some interviewees, more than one interview was conducted. For health care professionals, the interview guide explored La Virgen’s response to IPV, how it had been integrated in teamwork, individual differences and involvement, and relationships within the team, among other aspects. For external actors, the guide focused on their perceptions of the workings of the health care system in general and La Virgen in particular. Non-participatory observation was conducted during consultations of family doctors, nurses and the social worker, in waiting areas, at the women’s group meetings at the PHC facility, at the women’s group meetings in the community, and at monthly team meetings for discussing IPV and malaise cases. During the observation, notes were taken. The objective of these observations was to become familiar with the setting and to gather information regarding the organizational context in terms of how the team “ordinarily worked”, the group dynamics, the consultation dynamics, and the professionals’ attitudes and opinions regarding IPV. The observations pointed to short consultation times and heavy workload, and the role of the management style. It allowed clarification of how the women malaise approach was applied during medical consultations and in the women’s group gatherings. For ethical reasons we chose not to be present in any consultation with a woman exposed to IPV.

Within regional level institutions, information was collected to explore the wider contextual factors. Laws, health plans, protocols, training plans and other documents concerning IPV within the autonomous health system were reviewed. Three interviews were conducted with civil servants in charge of IPV at the level of management within Murcia’s health system.

Additionally, we assessed the responsiveness of the health professionals to IPV through the

Physicians Readiness to Manage Intimate Partner Violence questionnaire (PREMIS) [14, 25–27]. We also documented the discussions and consultations among professionals dealing with cases of IPV through a social network analysis questionnaire [28]. The analysis and findings from these questionnaires are presented in Additional file 1, but will not be further discussed here.

### Data analysis

The data analysis broadly consisted of two phases. First we set out to describe the case in terms of the intervention, its implementation, the actors, the processes, the outcomes, the context and the potential mechanisms. We used the propositions contained in the preliminary programme theory (PT1) as the main guidance to analyse the interview transcripts (verbatim transcribed and imported into Atlas.ti for data management and coding), as well as the notes taken during observation (also imported into Atlas.ti). We used a thematic analysis approach [29], whereby we started with predefined codes derived from the core elements of PT1 (e.g. team culture, team learning, response styles, adoption of protocols, training, etc.). However, relevant issues that were not predefined by PT1 emerged from the analysis, for instance the wide variation of professionals’ commitment due to personal motivation. Next, the codes and first aggregations were further explored and themes emerged.

The second stage of the qualitative data analysis consisted of searching for CMO configurations or patterns (demi-regularities) that provide an explanation for the observed outcomes [18, 19, 30]. This uses the retroduction approach, whereby the observed outcomes are explained by looking into the mechanisms and context elements. In our study, we brought together the qualitative information and the information from the questionnaires and the social network analysis (see on Additional file 1) to develop a thick description of the case (following the guide in the Additional file 2) and to identify CMO patterns.

During the development of the thick description, mechanisms emerged in the process of looking for explanations of the outcomes, and we found they were linked to specific contextual factors. Pattern matching was used, to a certain extent, in order to validate these CMO configurations [31] and alternative explanations were sought for. Triangulation was carried out by looking for confirmation of qualitative analysis findings in the quantitative data and by comparing analytical notes of the different researchers involved. For example, the contribution of the women malaise approach in driving a more comprehensive response to

IPV was ‘tested’ by checking the extent to which professionals who were not responding to IPV were implementing such an approach. Member checking was done during the first phases of the analysis, by discussing preliminary patterns with some of the professionals working in La Virgen, and through a presentation of our preliminary interpretations to a group of such professionals.

### Ethical considerations

Ethical approval for this study was granted by the Ethical Committee of the University of Alicante (Spain). The study was presented to the regional public health authorities, who approved its implementation. The study was presented to LV’s health care team and permission was later requested to conduct the study at LV. This meeting also served to explore improvements in the study design, such as the possibility of interviewing women who were users of the health services.

Written informed consent was sought from all the participants in the study prior to the interviews. Confidentiality was assured and pseudonyms were used for all respondents. We used a pseudonym for the primary care centre - La Virgen. Participants were informed that no individuals would be named in the presentation of the study’s results. In the case of interviews with women using the services, measures were taken to ensure their privacy and safety; they were first contacted through LV’s social worker, who only approached women no longer living in violent relationships and whom she considered were safe to interview.

The preliminary results were presented to the participants for member validation and their suggestions and comments have been included in the findings.

## Results

In this section, we present both mechanisms and important background information that was uncovered in the data collection and analysis process. Hedstrom and Swedberg’s classification of “macro-to-micro”, “micro-to-micro”, and “micro-to-macro” mechanisms has been particularly useful to organize the results [32, 33]. Micro-to-micro mechanisms refer to the way in which team learning processes were driven by the interaction of the team members with each other and with their organizational context. Macro-to-micro mechanisms refer to the way in which the implementation of national/regional interventions related to health care response to IPV triggers changes in the practices of the primary health care team through learning.

Finally, micro-to-macro mechanisms refer to the way in which team learning processes generate an array of new services and a style of responding to women exposed to IPV.

### Micro-to-micro: Capacity and interest in creating spaces for team learning, team work and therapeutic responses to IPV

We found that, in La Virgen, the existence of a group of committed and knowledgeable professionals together with a management style that respected and promoted professionals’ initiatives, contributed to the capacity and the motivation to create spaces for team learning, team work and therapeutic responses to women exposed to IPV. Contextual factors, mainly individual commitment to the issue, shaped the specific mechanisms triggered among different subgroups of professionals, as well as the outcomes achieved.

At La Virgen, there were different degrees of commitment to IPV. The professionals most committed to IPV - the social worker, three family doctors and two nurses - developed a particular style of responding to IPV. They have not created an institutional group, but they frequently discussed the cases among themselves. These professionals have developed a woman-centred IPV response that is aimed at responding to women’s needs, empowering women and improving their well-being. They were considered by the entire team as the most knowledgeable staff and they were most frequently consulted for cases of IPV. At the individual level, their interest in IPV emerged from their personal ideology - close to feminism and equality. This motivated them to further learn about the topic and to improve their own practices. The beneficial effects they perceived among their patients further reinforced their women-centred approach. The personal commitment of these professionals, supported by the support of the management team of La Virgen, triggered mechanisms of continuous self-learning on IPV, increased self-confidence related to responding to IPV and feeling nurtured within a group. All in all, these mechanisms enabled them to better respond to IPV:

‘*Here, we have gathered a group of people who believed they could work on these issues, who felt good by doing so, and who we believed could do it and share between us. This sharing has enriched us. And we have also seen that this way of working has having a beneficial effect on us and especially on our patients, and this is what I think is most valuable; I mean, I can use what I learn from my colleagues to improve the care I offer to my patients’* (Family doctor 6).

The result was a particular style of responding to women exposed to IPV, which included: 1) developing a trusting relationship with the woman, 2) working as a team, and 3) implementing the women malaise approach. The latter included paying much attention to detecting signs of malaise, exploring their roots, and offering therapeutic spaces to empower women and engage them to make decisions that contribute to improved well-being and health- including ending abusive relationships.

Our analysis found that this small group of highly committed professionals served as inspiration for others, both through their daily activities and engagement, and through the spaces they have created, such as the monthly meetings of the malaise group. This small group of committed professionals inspired their not-so-highly motivated colleagues by showing that a woman-centred approach was both effective and professionally satisfying. They provided ‘back up by experts’ and thus contributed to feelings of self-efficacy and self-confidence. This in turn stimulated them to participate in the group, and consequently enhance their knowledge and, to a certain extent, their practices:

*‘When I started working here, there was a different social worker, and we referred cases to her, but there wasn´t…, there wasn´t the kind of commitment that there is now. I think it is the two of them [the social worker and the IPV coordinator], they have been very active, and they are pulling us all along as well.’* (Medical coordinator)

The mechanisms of feeling inspired and backed (self-confidence and self-efficacy) was also triggered through the participation in the monthly team meetings of the “malaise group”, open to all health care professionals in La Virgen. The malaise group may also have triggered another key mechanism; deep involvement with IPV cases can easily affect a provider’s own well-being and could negatively influence the way these professionals respond to women suffering from IPV. The malaise group served in effect as a debriefing space, in which emotions, including anxiety and frustrations, were shared. This verbalisation of deep personal feelings helped staff to cope with the emotionally intense consultations and the resulting personal work-induced stress:

*‘We didn´t put limits on this woman, and this woman overwhelmed us emotionally. I remember that one Christmas, I was about to take this woman to my house, so stressed was I with the case. …And this case, we took to the malaise group, we discussed it and there was a before and after with this case…* ‘(Social worker - PHC team).

Recognition that the woman-centred approach was beneficial for IPV victims reinforced the providers’ motivation to implement a woman-centred response to IPV, because it contributed to sustained or increased self-efficacy and self-confidence. At the same time, providers expressed their uncertainties about whether this working style could be sustained if their working conditions were to worsen.

*‘This is the way I work, it feels good, and I think that I will never be able to forget that this is the way I work… But, who knows, if they continue increasing the number of patients, the workload, if they don’t give me time to rest, if for any training that I would like to attend I will have to do it in my free time…, I am not sure what I will do…’* (Family doctor 1 - PHC team).

This last quote illustrates how the working conditions may be an essential condition for an integrated response to IPV to be successful: staff may critically need the autonomy to organize their work and to create therapeutic spaces. The management at LV made this possible through respecting the health providers’ professional autonomy. For instance, management allowed the social worker and one family doctor to devote one morning per week to the women’s group. However, this conducive approach also meant that those who did not want to engage in responding to IPV were not forced to do so.

The fact that systems for referral of IPV victims to other services and facilities worked well further motivated an integrated IPV response on the part of providers, who felt that their efforts were supported and complemented by the response of other services (a mechanism of feeling connected and supported).

However, it has to be noted that not all health professionals in La Virgen have been inspired by this woman-centred approach. While there was a group of committed and knowledgeable team members, and a larger group of professionals who were interested and participated in the malaise group, others were much less involved in these activities. Two professionals were pointed out by others as being overtly resistant to IPV and having spoken negatively about the issue in public meetings and in informal spaces. This heterogeneity in involvement is clear in the large variation between the individual scores of the PREMIS questionnaires (Additional file 1). In terms of professions, midwifes seemed to be less involved, although reasons for this were not mentioned. Not all staff members were able or willing “to see” or detect the cases of IPV amongst their patients. Participants noticed that IPV cases were always detected “by the same people”, as the IPV coordinator stated:

*‘Many people do not want to get involved with this issue, do not want to deal with this issue, and I tell them: “if you happen to have a patient [exposed to IPV] and if you can’t or don’t want to deal with the problem, call me, o refer her to me. And they say: “Fine, fine, OK, OK”. But it doesn’t really work like that. IPV cases are always seen by the same people…*’ (IPV coordinator - PHC team).

Thus, the mechanisms previously mentioned of feeling backed, feeling inspired, feeling legitimated, etc., were not triggered in all health professionals in La Virgen, and personal ideology and motivation were key factors that influenced whether those mechanisms were triggered or not.

A key aspect of LV’s response to IPV, acknowledged and recognised as such by all the participants in this study was the “women’s group”. Even those providers who were not that much engaged in the IPV response knew about this activity and valued it, and many referred women to this group. For health professionals, being able to refer women with non-specific health complaints to the women’s group, and perceiving improvement in these patients, helped them feel more satisfied with their work (self-efficacy).

### Macro-to-micro: legitimisation of the work of committed professionals

Differentiating between a core group of committed staff and other staff members at La Virgen allows for identifying different mechanisms that influence team learning and IPV response. For committed staff, regional and national policies and programmes may not have had much influence on their response to IPV: these professionals were already addressing IPV cases through the women’s malaise approach. However, the policies did play a role in providing legitimacy for their work and were considered a strong sign of recognition (mechanisms of legitimisation and recognition). Being backed by a higher level of the system reassured these professionals when facing opposition from colleagues.

‘*Something that has been very important is that now the issue of IPV is taken seriously, there are policies, programmes, we are told by the regional administrators that this is something that we have to deal with, and I think that this has had an impact. I mean, we are no longer the “Quixote’s”, now we are in line with what the regional policies say…’* (IPV coordinator).

The policy also legitimated the function of the IPV care coordinator. Indeed, the official designation of a nurse as IPV coordinator (who was already committed and interested in IPV before the policy was implemented) increased the professionals’ involvement and response to IPV cases in two ways. It provided a physical point of reference with whom providers could consult, and it pushed family doctors and nurses dealing with victims of IPV referred from emergency units to act or to allow other professionals to act on their behalf. Thus, at least for women referred from emergency units, follow-up at the primary health care centre was not entirely dependent on the awareness of IPV of the doctor/nurse that she had been assigned.

*‘The first thing I do when I get the IPV reports from the hospital, or when a woman suffering*

*IPV comes directly to me, is to check who her family is doctor and who her nurse is. […] Because what I have to do is to ensure that there is somebody who supports this woman and accompanies her in the process. […] If they are in complete opposition, then I try to change the family doctor or nurse assigned to this woman.*’ (IPV coordinator, PHC team)

Professionals from LV mentioned having benefited from participating in training courses on IPV offered within the Regional Plan. Since participation in such courses was on a voluntary basis, it improved or refreshed the knowledge of those already (somehow) committed and interested. Thus, training programmes only triggered the mechanisms of self-efficacy through continuous self-learning among the professionals who were committed enough to attend such programmes:

*‘The existence of those courses allowed me to become trained…. It was something that I wanted to do. If I wouldn´t have been interested, I would not have attended the courses, even if they had been offered. I was interested and at the same time, I was offered the opportunity to participate.*’ (Family doctor 1- PHC team)

We found that other components of the regional IPV plan, such as guidelines and monitoring systems, were not important in shaping the response of the team at LV to IPV. For instance, the protocols may have served to back the efforts of committed providers by acknowledging and legitimising their responses to IPV, but the impact of the protocols in guiding health providers’ response was unclear, as one family doctor pointed out:

*‘Yes, politicians’ fever of protocols… At the end of the day, we clinicians have so many things to bear in mind. It is not possible to follow a flowchart for everything…, that’s crazy…*’ (Family doctor 2- PHC team)

Respondents did not the monitoring system as a positive influence on the LV’s response to IPV. Indeed, professionals expressed that monitoring focused on numbers, but not on support and motivation (mechanisms of feeling backed and legitimated not triggered).

*‘They want numbers, quantity, how many people we have seen. But, what about team work, quality? I don´t feel that we are heartened, encouraged, rewarded, I don’t see that… Maybe it´s being done, but I don´t see it.*’ (Social worker - PHC team).

For professionals who were not interested in the topic from the start, these external interventions had little impact on their praxis: since training activities were offered on a voluntary basis, they hardly participated, and since monitoring systems were weak, they did not feel the urge to do more. Thus the mechanisms of individual motivation to respond to IPV were not triggered among those professionals.

To conclude, the creation of learning spaces at the health centre and day-to-day contact with the committed professionals played a more important role when it came to motivating such professionals:

*‘Now, we see that it’s not just a matter of injuries. This is also a social problem and an important one, but I think this process has been mainly one of self-learning, I mean, colleagues who have prepared sessions, who have been interested in this issue, have told the rest of us…’* (Family doctor 5 - PHC team)

### Micro to macro: How La Virgen influences regional policies for IPV

During the interviews, it became clear LV had become a role model for Murcia, both from the point of view of those in charge at the regional health system and of the professionals working in other public institutions dealing with IPV, such as the municipal and regional services for IPV victims. La Virgen’s social worker and some of its family doctors participated in regional initiatives such as the “Technical Commission of the Gender Based Violence Training Plan”. They participated as trainers in workshops for health professionals, and in the development of the “Regional protocol for a health care response” and of Murcia’s Women’s Health Plan. By participating in these fora, LV’s model for IPV response extended beyond the confines of the health centre to influence service providers in other settings. Their participation at this level also triggered legitimation and recognition mechanisms.

LV’s model for IPV response was able to have an influence on regional policies because of a configuration of elements. First, the committed group of professionals of LV were eager to influence the regional health systems’ response to IPV (mechanism of professionals’ motivation to work beyond their clinical practice). Second, the group members proved to be experts in the matter, which gave them credibility in the eyes of their peers. Third, the Murcia Regional Health System IPV Programme managers involved service providers in the design of policies and programmes and thus created a context of inclusive decision-making processes, in which IPV professionals could operate as legitimate and respected experts.

‘*Currently, Eva [the social worker] and me, we are participating in the development of Murcia’s Women’s Integral Health Plan, which includes all the services that should be offered to women in Murcia’s Regional Health System. Eva managed to include an entire chapter on how to work with women’s groups within the primary health care teams, and there is a chapter on IPV detection.*’ (Family doctor 6).

## Discussion

This study found that the health care team at La Virgen has managed 1) to engage a number of staff members in actively responding to IPV, 2) to establish good coordination, mutual support and continuous learning processes related to IPV, 3) to establish adequate internal referrals within La Virgen, and 4) to establish good coordination and referral systems with other services.

We found that Murcia’s regional interventions aimed at implementing a health care response to IPV (protocols, training and monitoring system) have not equally reached all La Virgen´s professionals and that its adoption was mediated by the individual interest in IPV. Since training was offered on a voluntary basis, only those professionals with interest in the topic benefited from the training. The literature shows that health care providers who are well trained can improve referrals to specialist domestic violence agencies [8], but we did not find studies comparing compulsory training on IPV for health-care providers versus elective training. Programmes that introduce training on IPV as part of the compulsory curriculum of medical studies have showed promising results [34]. In Spain, regional managers in charge of implementing IPV training programs have divergent opinions on whether IPV training should be made compulsory for all health providers or not [22]. In regions where training on IPV has been compulsory, a higher percentage of providers have received basic training.

We found that the adequate detection of women suffering from IPV is a complex process that requires more than asking questions and following the steps of a protocol. Consequently, noncommitted professionals at La Virgen were not successfully detecting and referring patients to colleagues. This finding aligns with results from other studies that question the effectiveness of protocolised IPV screening programmes within primary health care facilities not backed by other actions [35–38]. La Virgen’s focus was not placed on IPV detection. Rather, it aimed at detecting and responding to less specific symptoms, called “women’s malaise”. The women’s malaise approach is grounded in feminism and states that the subordinate social position of women contributes to illness. Illness should thus be addressed through a gendered biopsychosocial approach [23, 24, 39], which combines a focus on gender with a personcentred approach to health. At its core, the person-centred approach draws attention to patients’ individual identities, in contrast with “illness-centred medicine”. The relationship between health professionals and the patient should be characterised by respect, coordination of care, high-quality communication and provision of information and patient involvement in decisions about care [40–44].

The case study of La Virgen indicates that the concept of women’s malaise can smooth team learning on how to respond to IPV and make such responses more comprehensive and woman-centred. An important number of women exposed to IPV complain from non-specific symptoms, and seek recurrent consultations from health professionals with little improvement [1, 3, 4]. The women’s malaise approach seeks to explore non-specific symptoms considering the social position of the woman in question. It can be especially helpful in cases where women exposed to IPV are not willing or capable of recognizing their situation as IPV [37, 45]. Thus, for health professionals, it might be easier to detect women’s malaise as compared to IPV. The fact that the women malaise approach makes explicit how gender inequalities affect women’s health and wellbeing may facilitates understanding IPV as a health problem in which health providers have a responsibility to act. In addition, the spaces generated by this approach and to facilitate its implementation (the professionals’ monthly meetings and the women´s group) can encourage heath professionals’ to respond to IPV.

In the case of La Virgen, the focus on women’s malaise allowed health professionals to better detect IPV. Being able to refer women with symptoms of malaise to the social worker and/or the women’s group supported La Virgen’s IPV response. This approach may be better aligned with the ongoing process of managing IPV than the protocolized detection- acute response- referral approach to violence. The latter may also negatively affect health providers’ response to IPV, since women’s relapses often bring about feelings of failure on the part of health professionals (despite the fact that relapses are a known part of the IPV cycle ) [46].

The case study of La Virgen points to the women malaise approach as a potentially relevant contextual factor shaping health-care team’s responses to IPV. We have not been able to find other studies linking such an approach with improved IPV detection and management. However, evaluations of such an approach in Spain provide evidence that support improvement on other health indicators such decreasing overutilization of services, reducing dependency on analgesics, and improving patients’ and providers’ satisfaction [23]. At the international level, similar approaches (i.e. the woman-centred approach in midwifery, or people centred care) appear to have positive effects on patients’ satisfaction with care, although the evidence is not conclusive [42–44]. The WHO clinical and policy guidelines on IPV strongly recommends the implementation of women-centred care to better respond to the needs of women exposed to IPV, although they also recognise that the quality of the evidence supporting this recommendation is only indirect, and even the definition of what is conveyed in such approach is vague [3].

Despite La Virgen’s achievements, our findings also point out that the type of care received by women exposed to IPV is strongly dependent on the individual characteristics of health professionals. This has been shown by other authors and points to the difficulties of sustaining and institutionalizing health care responses to sensitive non-biomedical issues, including IPV [7, 22, 47, 48].

Finally, it is interesting to point out the way in which successful experiences - such as La Virgen - can influence the practices of other primary health care teams. In the case of La Virgen, there was a group of committed professionals who were aware of IPV issues and eager to share knowledge and expertise with other professionals. The new policy legitimised their practices and created a structure for disseminating these practices to other health care teams. However, this study also shows that the influence of these channels (training programs, institutional plans) to change providers’ practices remains limited.

This analysis allows us to modify the initial programme and end up a PT2 that reads:

An intervention to improve the response to IPV within health care that consists of implementing evidence-based protocols and guidelines, training of health professionals, monitoring systems of policy implementation and installation of IPV coordinators within the PHC team is likely to reach different provider groups differently in function of their personal motivation (contextual factors at the individual level).

The existence of the intervention in itself (even if weakly implemented) provides legitimacy and an enabling structure for primary health care professionals already responding to IPV (mechanism for a sub-group of actors), and contributes to good practices and clinical outcomes. It has little impact on providers who are not committed to IPV care.

Personal attributes, including a sensibility to issues of IPV, often based on personal ideology feminism and/or equality - (contextual factors at individual level), facilitate uptake of the IPV policy. Team attributes that enable IPV responses included a woman-centred approach and a strong primary health care approach (contextual factors team level), while organizational attributes include a management style that values team learning, teamwork and individual initiative (contextual factors team-organization). Safe spaces for reflection and case discussion and the presence of experienced providers (contextual factors team) facilitate team learning and contribute to increased self-confidence of less experienced providers (mechanism). Therapeutic groups for women and adequate referral networks (contextual factors) also provide further support for professionals dealing with IPV, who felt that they have something useful to offer to these women (self-efficacy and self-confidence mechanisms).

This study has a number of limitations. We explored the reasons why certain health providers applied the women malaise approach and responded to IPV, i.e. personal ideology, considering it as rewarding. However, even if factors that hinder the implementation of such approach emerged, like time constrains or workload, we failed to inquiry the personal reasons why some providers did not engage in such approach.

The study of one only exceptional case allowed us to refine the initial PT1, but leaves many unanswered questions that deserve further exploration. However, this study is part of a larger evaluation in which 16 cases will be included in total, which allows us to refine the PT in a number of different contexts (4 different health centres in 4 regions). In line with the realist evaluation cycle, the results of this study are the starting point of a new evaluation cycle that will allow us to further refine the PT2.

Given the few published papers that document the process of developing and refining programme theories, this paper presents methodological guidance for authors using this approach in public health research.

This study struggled to distinguish between implementation failure and policy failure. However, as we pointed out above, the aim of this study was not to end up with a final middle range theory explaining why and how primary health-care teams learn to respond to IPV, but rather to come up with refined programme theory that could be a better starting point for future cycles of realist evaluations.

## Conclusion

The primary health care team of La Virgen is involved in a continuous learning process in terms of IPV response, even as the participation in this process varies between professionals. La Virgen has managed to create and sustain a process that promotes team learning on IPV and ensures an internal and external referral system to support providers dealing with IPV cases. This process has been backed, but not caused, by a favourable policy for integration of a health care response to IPV. Specific contextual factors of La Virgen, including the management style and the approaches to primary health care and women’s malaise facilitated the uptake of the policy.

To a certain extent, the experience of La Virgen has the potential to shape the IPV learning processes of other primary health care teams in Murcia. However, this potential is subject to the current political context of decreased social funding, less investment in primary health care compared to specialized care, and a reduced interest in IPV, which might make it more difficult to sustain and expand La Virgen’s successful model.

Finally, the aim of this study is not to end up with definitive answers but to identify potential mechanisms and contextual factors relevant in triggering (or hindering) the development of different health care responses to IPV, which can be ‘tested’ in subsequent case studies. This is in line with the cyclic nature of realist evaluation, where emerging programme theories become the starting point of a new cycle of realist evaluation. The contribution of the women malaise approach, individual motivation, professional background, organizational context, etc. to a primary health care team’s response to IPV needs to be further tested. This could be attempted through exploring its relevance in other purposively selected cases.

## Additional files

Additional file 1:
**Data collection and main findings from the application of the PREMIS and the Social Network Analysis questionnaires.**
Additional file 2:
**Guide for the thick description of the case.**
